# Fast microglial activation after severe traumatic brain injuries

**DOI:** 10.1007/s00414-020-02308-x

**Published:** 2020-05-05

**Authors:** Julia Lier, Benjamin Ondruschka, Ingo Bechmann, Jan Dreßler

**Affiliations:** 1 Institute of Anatomy, University of Leipzig, Liebigstraße 13, D-04103 Leipzig, Germany; 2 Institute of Legal Medicine, University of Leipzig, Johannisallee 28, D-04103 Leipzig, Germany

**Keywords:** Chronic traumatic encephalopathy, Immunohistochemistry, Inflammation, Microglia, Traumatic brain injury

## Abstract

Traumatic brain injury is among the leading causes of death in individuals under 45 years of age. However, since trauma mechanisms and survival times differ enormously, the exact mechanisms leading to the primary and secondary injury and eventually to death after traumatic brain injury (TBI) remain unclear. Several studies showed the versatile functions of microglia, the innate macrophages of the brain, following a TBI. Earlier being characterized as rather neurotoxic, neuroprotective capacities were recently demonstrated, therefore, making microglia one of the key players following TBI. Especially in cases with only short survival times, immediate microglial reactions are of great forensic interest in questions of wound age estimation. Using standardized immunohistochemical methods, we examined 8 cases which died causatively of TBI with survival times between minutes and 7 days and 5 control cases with cardiovascular failure as the cause of death to determine acute changes in microglial morphology and antigen expression after TBI. In this pilot study, we detected highly localized changes in microglial morphology already early after traumatic damage, e.g., activated microglia and phagocyted erythrocytes in the contusion areas in cases with minute survival. Furthermore, an altered antigen expression was observed with increasing trauma wound age, showing similar effects like earlier transcriptomic studies. There is minute data on the direct impact of shear forces on microglial morphology. We were able to show localization-depending effects on microglial morphology causing localized dystrophy and adjacent activation. While rodent studies are widespread, they fail to mimic the exact mechanisms in human TBI response. Therefore, more studies focusing on cadaveric samples need to follow to thoroughly define the mechanisms leading to cell destruction and eventually evaluate their forensic value.

**Electronic supplementary material:**

The online version of this article (10.1007/s00414-020-02308-x) contains supplementary material, which is available to authorized users.

## Introduction

Traumatic brain injuries (TBI) have a biphasic course [1]. While tissue destruction and necrosis occur immediately and directly at the impact zone together with intracranial or intracerebral hemorrhages as well as cortical contusions (primary phase), numerous changes and pathways in inflammation, edema, and blood flow disturbances start subsequently within minutes, hours, or days due to pathophysiological regulatory processes of the brain tissue. These mechanisms are usually summarized as the secondary injury [2, 3]. Due to the heterogeneity of the initial trauma, a reconstruction of the precise events leading from primary to secondary injury is very difficult. Therefore, the exact molecular and cellular mechanisms at acute, subacute, or chronic survival times after TBI remain to be defined in more detail [1, 3]. Given the high numbers of traumatic fatalities including head injuries worldwide, diagnosing the fatal injuries and estimating the TBI survival time are the main aspects of legal medicine and, therefore, were the aim of many forensic studies before [4–8]. Microglial cells, the resident macrophages of the brain, are activated in the course of traumatic brain injuries, producing cytokines and causing an influx of peripheral immune cells [1, 9]. While being a silent observer surveying the brain parenchyma in their ramified phenotype, microglia can target focal brain injury within minutes by projecting their processes towards the sites of damage, subsequently followed by alterations in morphology and protein expression [10, 11]. Histological studies in humans suggest that microglia activation can be sustained for many years after TBI [12], and their changes are therefore attributed to chronic traumatic encephalopathy stages; however, minute data are available for acute microglial changes in traumatized human brain tissue.

The verification of a TBI during the autopsy is relatively easy when severe intracerebral injuries are found. However, especially with only short survival times or in cases with severe destructions due to polytrauma, these findings are not necessarily established macroscopically. Since reliable and unique biomarkers of traumatic damage to the central nervous system (CNS) are still lacking despite years of active research [5, 13, 14], a closer look and detailed evaluation of immediate microglial reactions are of great forensic interest. Additional knowledge about time-dependent microglial changes after TBI may be used for a determination of trauma severity, survival time estimation, and a deeper understanding of mechanisms of traumatic death cases.

## Materials and methods

Brain tissue samples from the pericontusional zones, macroscopically unaffected areas of the contralateral cortex, and the cerebellum were taken from eight individuals, who died causatively after suffering a TBI, and five control cases during forensic autopsies, which are displayed in detail in Table 1. If the cerebellum was traumatized by contusions, also intracerebellar controls were taken. None of these cases suffered from any clinically relevant neurodegenerative disease, either known from the medical history of the deceased or autopsy results. No brain tissue sample showed signs of putrefaction.

**Table 1:** Characteristics of all cases used in this study

Case no.	Gender	Age	Trauma mechanism	Trauma type	Cortical contusion	Survival time	Cause of death	Brain weight	Degree of brain edema	PMI
Traumatic brain injury cases (*n* = 8)										
1	m	18	Car accident	Blunt force (impact)	Pons	Few min	Pons dehiscence	1380 g	None	44 h
2	m	32	High height fall	Blunt force (fall)	Cerebellum	10 min	Subarachnoidal hemorrhage	1100 g	None	36 h
3	w	73	Falling branch	Blunt force (hit)	Parietal	27 min	Opened TBI	1230 g	None	44 h
4	w	53	Car accident	Blunt force (impact)	Temporal	38 min	Blood aspiration	1280 g	Moderate	72 h
5	m	58	Motorcycle accident	Blunt force (fall)	Temporal	9.5 h	Brain edema	1570 g	Severe	35 h
6	w	73	Pedestrian accident	Blunt force (impact)	Parietal	2 d	Brain edema	1400 g	Severe	48 h
7	m	30	Motorcycle accident	Blunt force (impact)	Temporal	2.8 d	Brain edema	1610 g	Severe	46 h
8	w	78	Tram accident	Blunt force (fall)	Temporal	7 d	Multi-organ failure	1260 g	None	64 h
Control cases (*n* = 5)										
9	m	42				None	Acute myocardial infarction	1310 g	Moderate	89 h
10	m	67				None	Acute myocardial infarction	1250 g	None	94 h
11	m	47				None	Ruptured aortic aneurysm	1580 g	Severe	48 h
12	w	27				None	Sudden cardiac death	1410 g	Moderate	93 h
13	m	50				None	Acute myocardial infarction	1390 g	Moderate	40 h

Before embedding in paraffin, the tissues were fixed in a 4% aqueous solution of formaldehyde. Using serial sections (18 μm), we performed Nissl and hematoxylin eosin (HE) staining for routine evaluation, as well as immunohistochemistry for a variety of microglia/macrophage markers (Supplementary Table 1). Furthermore, we examined the occurrence of tau and beta-amyloid using the corresponding antibodies **(**Supplementary Table 1).

Antigen retrieval was achieved by using microwave pretreatment in citrate buffer (95 °C, 5 min). Sections were then treated with 1.5% H_2_O_2_ in methanol for 20 min. Blocking the tissue with 1% bovine serum albumin (BSA; A7906, Sigma-Aldrich, St. Louis, USA) in PBS/Triton (0.03%, PBS-T) prevented further nonspecific binding. Subsequently, primary antibodies were incubated overnight at 4 °C in the adequate dilution in 0.5% BSA in PBS-T. After rinsing with PBS-T, application of the biotinylated secondary antibodies followed, and the slices were incubated for 90 min at room temperature (1:100, Supplementary Table 1).

Slices were then treated with peroxidase (ExtrAvidin-Peroxidase; E2886, Sigma-Aldrich). Its activity was developed using 3,3′ diaminobenzidine tetrahydrochloride (DAB; D5905, Sigma-Aldrich) as a chromogen. After counterstaining using hematoxylin, sections were washed, dehydrated, and mounted.

To verify the antibodies specificity and to eliminate unspecific staining, positive and negative controls were made constantly for the staining charges.

## Results

This study detected clear changes in microglial morphology and expression profiles in TBI fatalities with increased survival times compared to control cases of fatal cardiovascular diseases, starting already after a short period between trauma and death.

The brain samples of all control cases showed comparable ramified morphology without signs of microglial activation (Supplementary Table 2). Furthermore, microglial cells appeared to be evenly distributed in the brain samples of cardiovascular fatalities.

### Early and highly localized activation of microglia

**Fig. 1:**
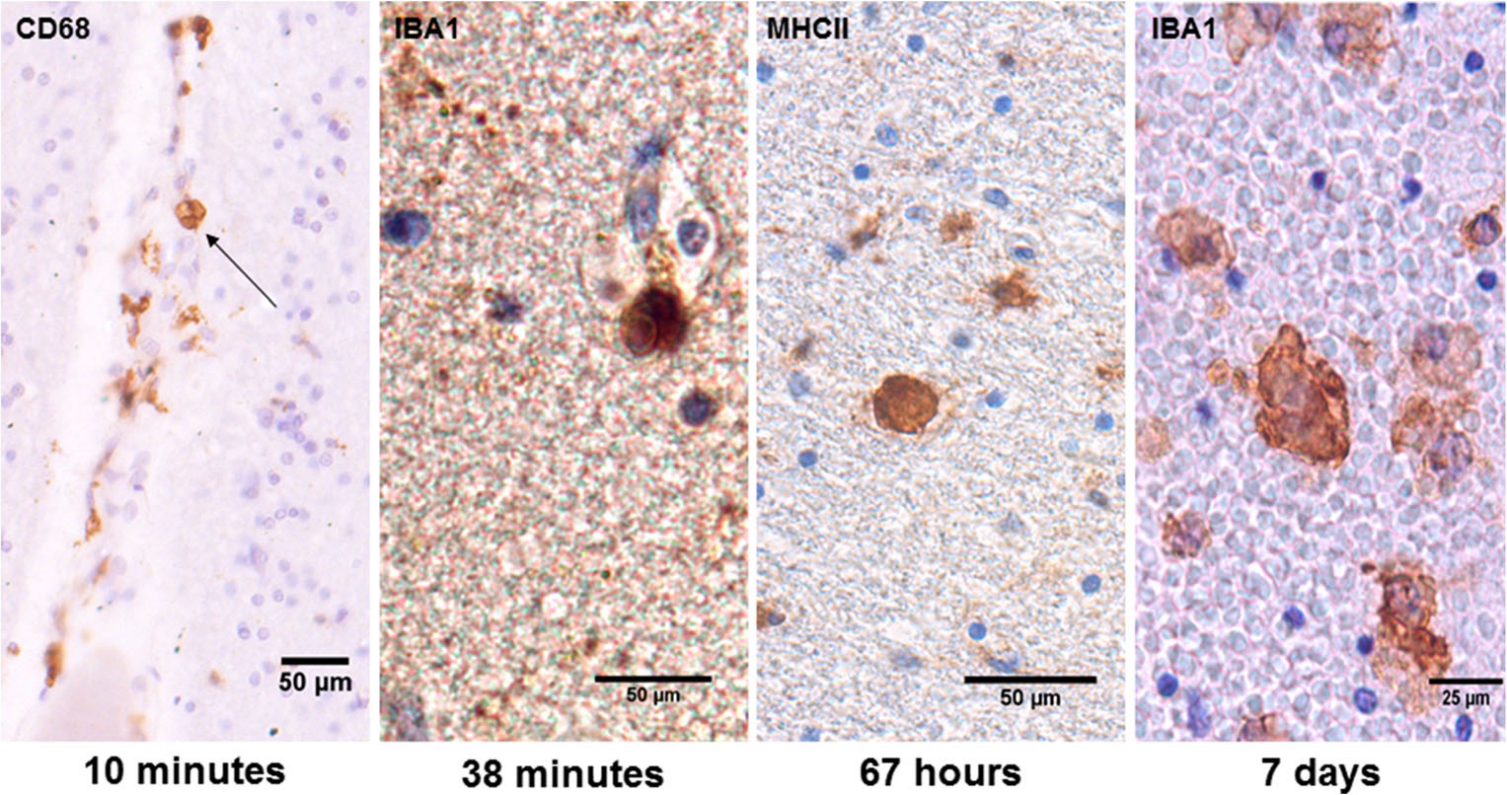
Hemophagocytosis by microglial cells in traumatic brain injury cases with different survival times. Already in cases with minute trauma survival, red blood cells are phagocytosed by microglia/macrophages in the contusion zones. This phenomenon seemingly appeared more frequently with increasing survival time and expansion of bleeding but stayed continuously inside the damaged brain area

In cases with short survival times (less than 1 h), microglial activation was already visible, and red blood cells were found to be phagocytosed by microglia within the directly affected area (Fig. 1). This phenomenon was detectable in survival times of 10 min at the earliest. Already in short distance to the contusion area, the so-called pericontusional zone, microglial morphology stayed ramified and seemingly unaffected.

By examination of sections deriving from contusion areas and their contralateral hemispheres, we detected that in cases with longer survival times (at a minimum 9 h), microglial morphology appeared to be similar within both brain hemispheres, mostly independent from their distance to the cortical contusion.

### Change of microglial protein expression

**Fig. 2:**
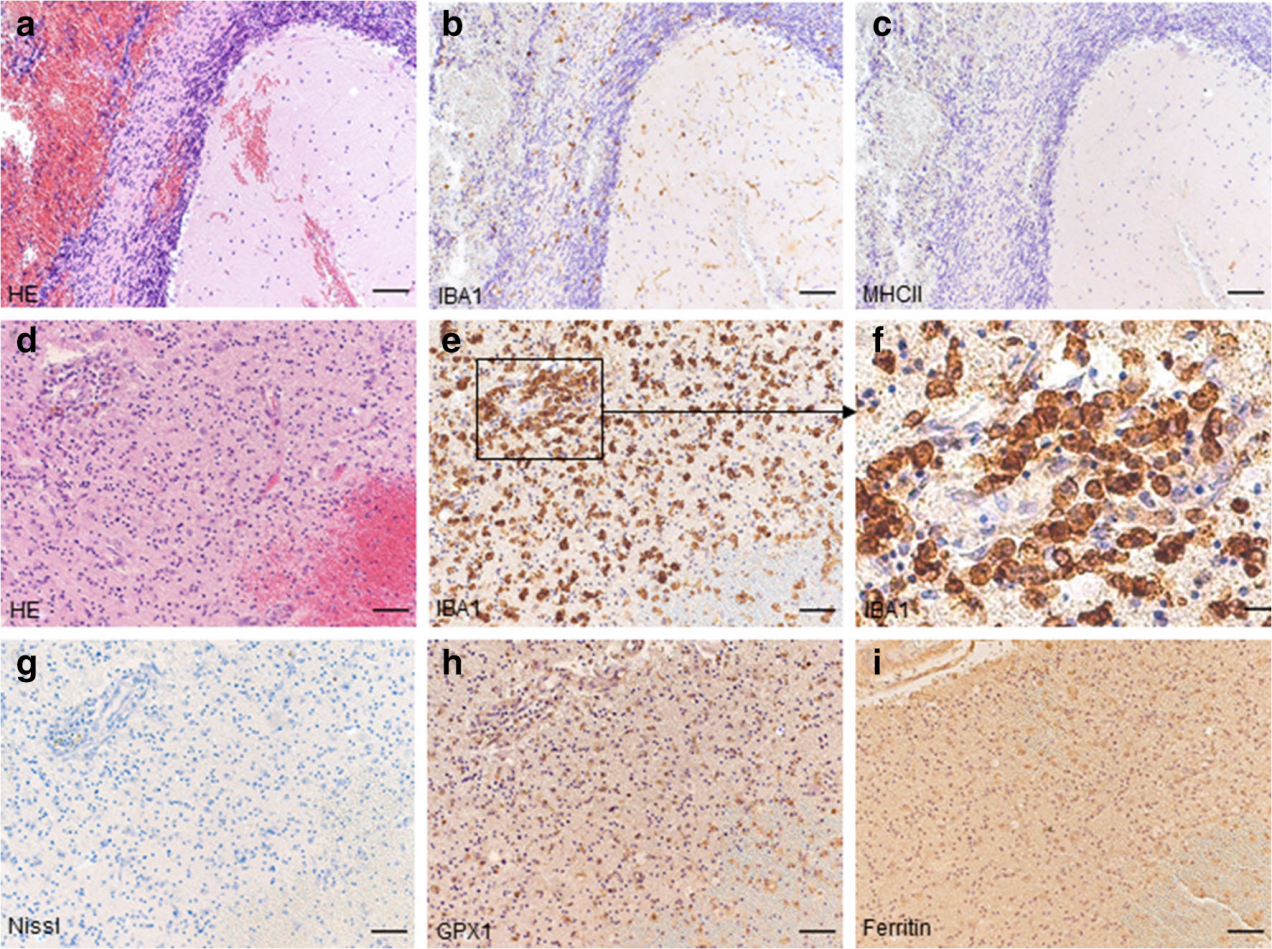
Comparison of microglial antibody expression after different trauma survival times. While IBA1 is expressed in a lethal traumatic brain injury case with a survival time of 10 min, showing activated microglia in acute vicinity of lesion and ramified cells in adjacent areas, MHCII immunostaining stayed negative (**a**–**c**, cerebellar contusion). Another fatal head injury with a survival time of 7 days showed changes in microglial morphology, migrating macrophages, and also altered antigen expression (**d**–**i**, frontal contusion). Scale bars **a**–**e**; **g**–**i**, 100 μm; **f**, 25 μm

In cases with shorter survival times (at the earliest in a case with 10 min survival), we detected morphologically activated microglia expressing IBA1 in acute vicinity to the traumatic lesions, while MHCII stayed negative in these areas (Fig. 2a–c). The case with the longest survival time (7 days) was characterized by meaningful changes in microglial morphology and an altered antigen expression. In this case, a positive staining for GPX1 and ferritin mirrored alterations in the transcriptional profile of microglia (Fig. 2d–i, Supplementary Table 2).

### Microglial activation and dystrophy due to physical forces

**Fig. 3:**
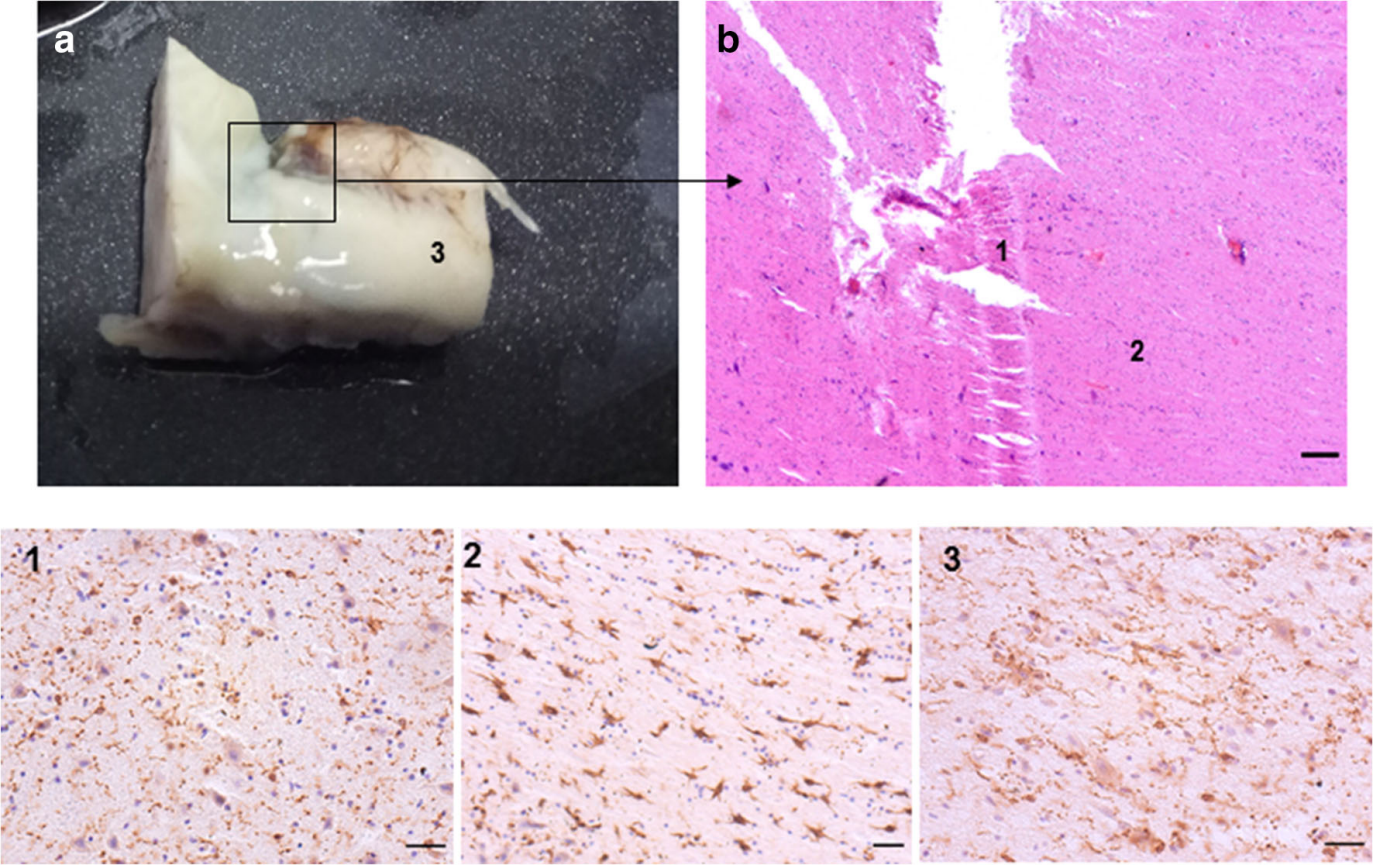
Shear forces have direct impact on microglial morphology. The pons was ruptured incompletely during an accident causing immediate death (**a**, macroscopic, and **b**, in hematoxylin eosin staining, scale bar 250 μm). (1) IBA1 immunostaining of microglia in immediate surrounding of the tissue dehiscence show pronounced dystrophy. (2) In close vicinity to the damaged area, microglial activation appears, characterized by thickened somata and shortened processes. (3) In short distance, microglia present a ramified morphology. Scale bars 1–3: 50 μm

In one case, the pons was ruptured incompletely in an accident (Fig. 3a) with death occurring within minutes. The HE staining showed tissue rupture and injuries caused by shear forces (Fig. 3b). IBA1-stainings of microglia in immediate surroundings of the tissue damage showed pronounced dystrophy, while in close vicinity microglial activation, characterized by thickened somata and shortened processes, already appeared. However, these effects were also within a restricted area, since a shorter distance away, microglia presented a ramified morphology (Fig. 3(1–3)), comparable to the presentation in unaffected and non-traumatized tissue.

### No fast deposition of tau or beta-amyloid associated with contusion

Several studies have linked the appearance of TBI with the development of tau and amyloid depositions and therefore the development of neurodegenerative injuries [15]. Given that human studies were able to detect axonal tau depositions within 24 h post-injury [16], the here presented cases were examined for the occurrence of such deposits. There was not a single accumulation of tau or amyloid in the vicinity to contusion areas in the survival timeframe investigated between minutes and 7 days.

## Discussion

This pilot study aimed to investigate the initial changes of microglial cells surrounding cortical contusions and in hypoxia-sensible areas of the brain to get a deeper understanding of the acute microglial activation and typical distribution patterns in the contusion area and the pericontusional zones of a TBI. The main results indicate a certain microglial morphology depending on severity, trauma mechanism, and survival time. These microglial changes could not only be easily examined with standard histological and immunohistochemical methods but also be used for daily forensic wound age estimation of TBI. A transformation in microglial protein expression towards a more reactive phenotype was also shown in transcriptional studies in rodents after a post-traumatic time course of 2 to 14 days [17].

Considering the biphasic course in the pathophysiology of TBI, microglia were shown to play a multifaceted role in the mechanisms following a trauma to the head. The depletion of microglia was shown to attenuate dendritic spine loss and neuronal apoptosis in the acute stage of TBI [18], therefore suggesting an active role of microglia in the early TBI pathophysiology. However, participation in repairing mechanisms [17] and maintaining the integrity of CNS barrier structures in dependence of purinergic receptors, e.g., the P2Y_12_R [19, 20], is one of the many neuroprotective capabilities. In future studies, especially the role of P2Y_12_R needs to be examined thoroughly, as it was characterized as a highly specific microglial marker, which is less expressed in the activated state [21]. Own research on purinergic signaling in TBI cases indicate a significant role of such receptors not only on microglia but also in many cell compartments in the TBI pathways [22]. The continuation of these receptor type characterizations showed potential for neuroprotective therapies (yet unpublished data).

While macrophages can maintain their metabolism also in a hypoxic environment [23], microglia are highly dependent on the aerobic generation of ATP to sustain their functioning [24]. However, in our cases, changes in microglial morphology appeared already after very short survival times. Therefore, migration of microglia and their phagocytic activity seems to be possible without an adequate oxygen supply for a certain amount of time in the agony phase of the death process, where all cell types are confronted with a generalized hypoxic situation. Thus, one needs to consider the possibility of supravital reactions within the brain tissue and to further evaluate microglial capacities in a hypoxic penumbra. Due to putrefactive changes being an exclusion criterium, autolysis was not a confounding aspect of such staining results.

Given the different microglial morphology within the area of interest, the authors consider the reaction on physical powers such as shear forces as a direct impact on the microglial morphology and, therefore, as a “proof of live” sign rather than agonal changes alone. The alterations have a certain configuration, which might mirror the severity and mechanism of the injury allowing for a conclusion on the extent of physical injuries. Furthermore, even severe brain edema did not seem to affect microglial morphology in our control cases to a relevant extent, supporting our hypothesis of trauma-induced microglial changes.

However, the extent of microglial capacities after the physical death needs to be examined thoroughly in the future, to correctly interpret the traumatic findings after TBI and to differentiate them from cell changes accompanying the death progress.

Due to a large heterogeneity and the complex pathogenesis, microglial TBI studies in humans are rare. However, rodent studies fail to mimic the exact mechanisms since any model can fully recapitulate the entirety of mechanisms leading to primary and secondary injury, as well as the diversity of injury mechanisms in human TBI [25]. The potential link to neurodegenerative diseases via tau or amyloid deposits as a response to TBI could not be proven with the cases investigated here. Therefore, the authors propose that the changes leading to these deposits might rather mirror (i) long-term effects of TBI in survival times longer than 7 days, (ii) repeated traumatism, or (iii) unspecific deposits with no pathological/traumatological correlation.

In the future, more studies with higher case numbers and tighter intervals between the survival time categories are needed to further obtain profound knowledge of microglial morphology and mechanisms. Then, the characterization and interpretation of microglial changes after TBI are useful as informative indicators on TBI response, not only to achieve an applicability of microglial morphology in forensic TBI analysis but also to detect possible targets for clinical (“neuroprotective”) treatment.

## Electronic supplementary material

ESM 1 (PDF 138 kb).
