# Supplementary material for: Fast microglial activation after severe traumatic brain injuries
Source: Int J Legal Med. 2020 May 5;134(6):2187–93. doi: 10.1007/s00414-020-02308-x (PMC7578125; doi:10.1007/s00414-020-02308-x)
Supplement: Supplementary file 1 — (PDF 138 kb). [file 414_2020_2308_MOESM1_ESM.pdf]

Supplemental material to:

Fast microglial activation after severe traumatic brain injuries

Julia Lier [1*], Benjamin Ondruschka [2*,#], Ingo Bechmann [1], Jan Dreßler [2]

[1] Institute of Anatomy, University of Leipzig, Liebigstraße 13, D-04103 Leipzig

[2] Institute of Legal Medicine, University of Leipzig, Johannisallee 28, D-04103 Leipzig

* These authors contributed equally to this work as first authors.

# Corresponding author

Corresponding Author:
PD Dr. med. Benjamin Ondruschka
University of Leipzig
Institute of Legal Medicine
Johannisallee 28
04103 Leipzig
Email: benjamin.ondruschka@medizin.uni-leipzig.de
Phone: (+49) 341 9715152

Supplemental Table 1: Antibodies used in the study.

**Antibody**	**Dilution**	**Host**	**Company (headquarter, country)**	**Catalogue number**
Primary antibodies				
Ferritin	1:300	rabbit	Sigma-Aldrich (St. Louis, USA)	F6136
Iba-1	1:400	rabbit	Synaptic Systems (Göttingen, Germany)	234 003
MHCII	1:300	mouse	Agilent Dako (Santa Clara, USA)	M0775
GPX1	1:80	goat	R&D Systems (Minneapolis, USA)	AF3798
CD68	1:300	mouse	Agilent Dako (Santa Clara, USA)	M0876
AT-8	1:500	mouse	Thermo Fisher Scientific (Waltham, USA)	MN1020
Beta-Amyloid	1:500	mouse	Sigma-Aldrich (St. Louis, USA)	A8354
Secondary antibodies				
Anti-rabbit	1:100	goat	Vector Laboratories (Burlingame,USA)	BA-1000
Anti-mouse	1:100	goat	Sigma-Aldrich (St. Louis, USA)	B7264
Anti-goat	1:100	rabbit	Sigma-Aldrich (St. Louis, USA)	B7014

Supplemental Table 2: Overview on staining results in all cases.

Case No.	Microglial morphology		IBA1		MHCII		GPX1	CD68	Ferritin	HE / Nissl
	Contusion	Control area	Contusion	Control area	Contusion	Control area				
**Traumatic Brain Injury Cases (n=8)**										
1	Dystrophic, activated	Ramified	+++	+++	+	-	-	-	-	Tissue rupture visible
2	Activated, apparent migration movement towards lesion	Ramified	++	++	-	-	-	+	-	Few intraparenchymal hemorrhages
3	Activated, dystrophic	Ramified, activated	+++	+++	++-+++	++	-	+ (perivascular)	+	Multiple intraparenchymal hemorrhages
4	Activated, partially amoeboid, dystrophic	Ramified	+++	++	+++	+	-	++	-	Few intraparenchymal hemorrhages
5	Activated, dystrophic, partially ramified	Ramified	++	++	++	+++	-	-	-	Severe intraparenchymal hemorrhages
6	Activated, partially amoeboid	Ramified	++	++	+	-	-	-	-	Intraparenchymal hemorrhages, dilated capillaries
7	Activated, ramified	Ramified	+	++	+++	++	-	-	-	Severe brain edema
8	Activated, amoeboid	Ramified, activated	+++	++	++	-	++	++	+	Pronounced hemorrhage
**Control cases (n=5)**										
9		Ramified, partially dystrophic		+		+ (perivascular)	-	+ (perivascular)	-	w/o hemorrhages
10		Ramified		++		-	-	+	-	w/o hemorrhages
11		Ramified		++		+ (perivascular)	-	-	-	w/o hemorrhages
12		Ramified		++		+ (perivascular)	-	-	-	w/o hemorrhages
13		Ramified, partially dystrophic		++		+ (perivascular)	-	+ (perivascular)	-	w/o hemorrhages
